# Ten simple rules for maximizing the recommendations of the NIH data management and sharing plan

**DOI:** 10.1371/journal.pcbi.1010397

**Published:** 2022-08-03

**Authors:** Sara Gonzales, Matthew B. Carson, Kristi Holmes

**Affiliations:** 1 Galter Health Sciences Library & Learning Center, Northwestern University Feinberg School of Medicine, Chicago, Illinois, United States of America; 2 Department of Preventive Medicine (Health and Biomedical Informatics), Northwestern University Feinberg School of Medicine, Chicago, Illinois, United States of America; Dassault Systemes BIOVIA, UNITED STATES

## Abstract

The National Institutes of Health (NIH) Policy for Data Management and Sharing (DMS Policy) recognizes the NIH’s role as a key steward of United States biomedical research and information and seeks to enhance that stewardship through systematic recommendations for the preservation and sharing of research data generated by funded projects. The policy is effective as of January 2023. The recommendations include a requirement for the submission of a Data Management and Sharing Plan (DMSP) with funding applications, and while no strict template was provided, the NIH has released supplemental draft guidance on elements to consider when developing a plan. This article provides 10 key recommendations for creating a DMSP that is both maximally compliant and effective.

## Introduction

Clinical and translational researchers have been aware of the increasing data management requirements of the National Institutes of Health (NIH) since its initial release of policies for data management and sharing in 2003 [1]. The initial requirement of submission of a data sharing plan applied to funding applications of $500,000 or more in direct costs per year and has evolved over the years in order to accommodate the nuances of managing clinical data, as well as increasing sophistication of research data management. After releasing a new Draft Data Management and Sharing (DMS) Policy and Supplemental Draft Guidance for comment in November 2019 [2], the NIH incorporated feedback from the community to produce the Final DMS Policy in October 2020 [3]. The Final DMS Policy requires a 1- to 2-page Data Management and Sharing Plan (DMSP) to be submitted with the application for all funded research. The intent of the Policy is to encourage data sharing to the extent that it is possible, as the policy states, the NIH expects that *“researchers are prospectively planning for data sharing*, *which we anticipate will increasingly lead researchers to integrate data sharing into the routine conduct of research*. *Accordingly*, *we have included in the final DMS Policy an expectation that researchers will maximize appropriate data sharing when developing Plans”* [3].

Sharing research data securely and efficiently is a key step toward supporting and advancing translational science, as it allows for savings in researcher time and effort and greater assurance of reproducibility. Concerns with research replicability and reproducibility lie behind the NIH’s guidelines and have been documented in regards to the larger research community extensively in the literature [4–6]. Open science practices, including publication of protocols and sharing of code, go a long way toward enabling research reproducibility. Sharing of the de-identified data from clinical studies, when possible, is also a crucial step.

Data sharing on the level required by the new policy is not new to researchers in certain fields, such as those familiar with the NIH Genomic Data Sharing Policy [7], the Model Organism Sharing Policy [8], and other existing sharing policies in the clinical research sphere where NIH funding is involved [9]. The update to existing practices required by the new policy is the requirement of submission of a DMSP with *all* NIH-funded research submissions, with an expectation of compliance and adherence to the plan (with allowances made for updates) throughout the lifecycle of funded projects.

**Fig 1:**
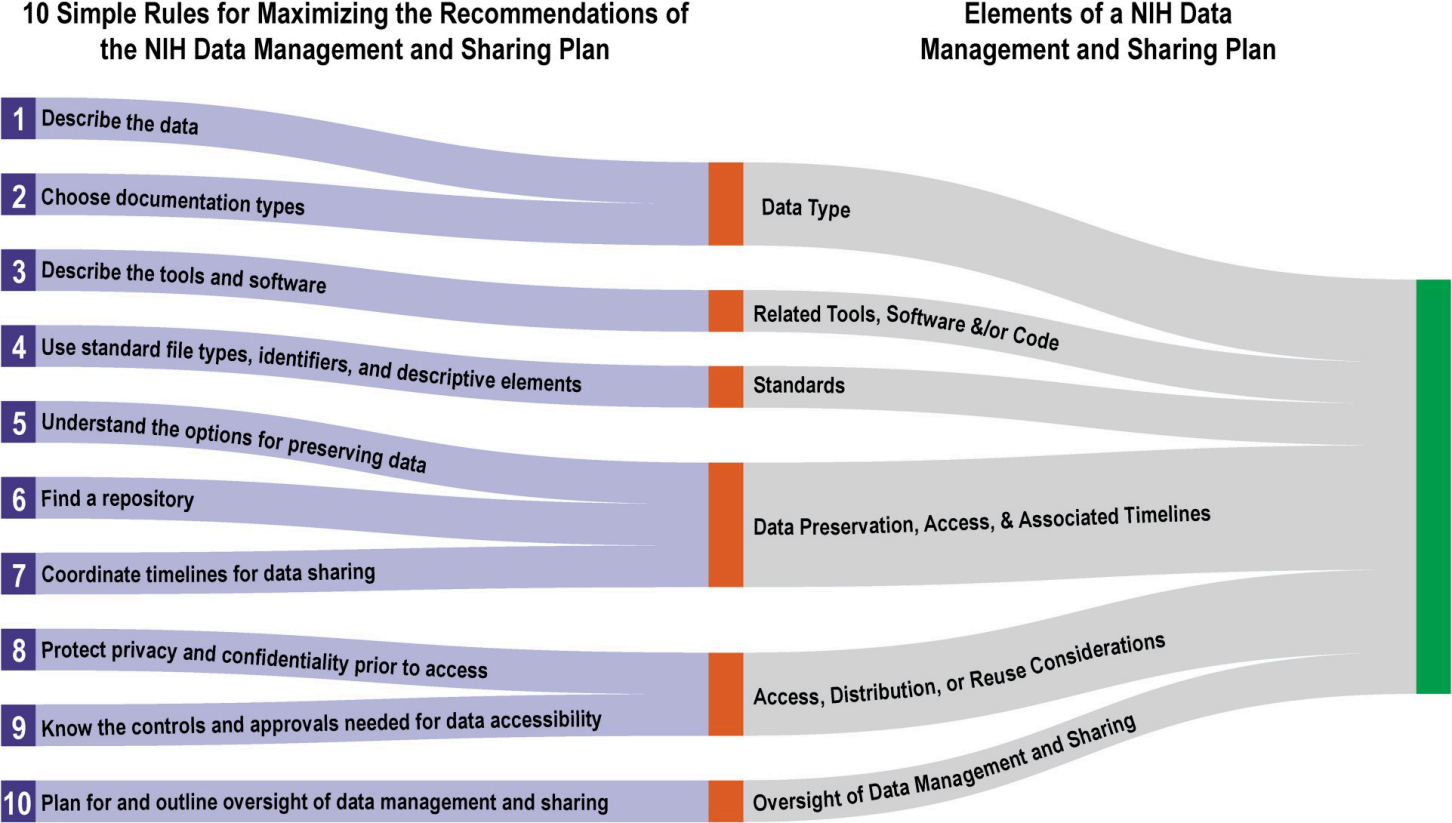
The 10 Simple Rules for Maximizing the Recommendations of the NIH Data Management and Sharing Plan mapped against the Elements of an NIH Data Management and Sharing Plan. This mapping shows how the rules in this article map to the recommended elements of an NIH Data Management and Sharing Plan as defined in the *Supplemental Information to the NIH Policy for Data Management and Sharing*: *Elements of an NIH Data Management and Sharing Plan*.

The 10 simple rules below are intended to assist researchers in both writing a plan that is compliant with the new data management and sharing requirements and that is maximized for incorporating as seamlessly as possible into research workflows. The rules are ordered as they pertain to the sections of the *Elements of an NIH Data Management and Sharing Plan* (DMSP Elements), the NIH’s supplemental guidance document on creating a data management and sharing plan, to demonstrate practical ways to meet the requirements (Fig 1).

### Rule 1: Describe the data: What is it, how much will be generated, and what is the level of processing?

#### Corresponds to DMSP Elements: “Data Types,” point 1

The DMSP Elements guidance requires description of the types of data that will be generated in the course of the project, including information about the data’s modality, level of aggregation, and level of processing [10]. Though the project is not yet begun at the time of the DMSP submission (which accompanies the budget justification in the grant application), list the data types the research team anticipates will be created. This can be addressed by the following:

- Modality (or high-level category): list the overall type of data to be created, such as genomic, imaging, text sequences, modeling data, etc.
- Formats: list the anticipated data formats to be created, such as CSV, TSV, XML, JSON, fMRI files, SAV, SAS, DTA.
- Amount: to the extent possible, list the number of files expected to be generated and/or their anticipated storage space (terabytes of data, petabytes, etc.).
- Aggregation: list whether individual or aggregated data provides insights into the research question(s) and also which type (aggregated or non-aggregated) will be shared.
- Processing: list the anticipated level of processing that will be pursued in the project and also the processing level of data that will be shared.

Regarding the portions of project data that may be shared, as referenced above, keep in mind that sharing of all data from the project is not required. Subsets of the full dataset may be shared based on what is legally and ethically permitted for sharing (more on this in rules to follow). Subsets can include portions of the data demonstrating the principles outlined in a resulting publication, small representative de-identified subsets, subsets allowing replication of the study, etc.

### Rule 2: Choose documentation types from the beginning of the project

#### Corresponds to DMSP Elements: “Data Types,” point 2

The NIH’s DMSP Elements requires that, in addition to describing the project data that will be produced, a description of the portion of project data that will be preserved and shared is required [10]. Though the project has yet to formally begin, the research team may already have in mind such categories of data, as well as the metadata descriptions that will accompany data throughout its lifecycle, and the types of documentation that will be employed in the project to keep track of the data. Though detailed documentation examples are not required at the time of submission (and would be too lengthy for a 1- to 2-page data management and sharing plan), it is a good time to consider the documentation that will be used in the project, which may consist of:

- **Metadata documentation:** explain whether the project will describe data using metadata such as the NIH Common Data Elements [11], the MIAME or MINSEQE [12] standards, or other metadata vocabularies that can be found through resources such as the Digital Curation Centre (DCC) [13].
- **Data dictionary:** A data dictionary describes aspects of the data at the most granular level. This document is generally maintained in spreadsheet form and outlines details of each variable, including both human readable and “coded” names, definitions, units of measurement, data types and ranges allowed, and permissible null values [14].
- **README files:** A README contains detailed information about data file formats, as well as data collection methodology, including details on instruments and software used, explanations of relationships between files, and details on quality control practices [15]. The format is generally a brief explanatory document outlining dataset structures, terminology, and definitions that make research data files easier to understand for secondary users, regardless of where these files are stored.

The abovementioned files will be helpful to have in later stages of the project, enabling compliance when the data-sharing stage nears. For any data that is not planned to be preserved and shared online for legal, ethical, or other reasons, a rationale is requested in the DMSP. Having such descriptive metadata providing general information on the content of the files can assist with reinforcing such rationales. In such cases, the types of descriptive files outlined above can serve to represent sensitive datasets without divulging protected information. Moreover, these descriptive files can be made available and discoverable through an institutional, generalist, or discipline-specific repository, with metadata denoting the location of the data and more detailed information about brokering access and use of the data.

### Rule 3: Describe the tools and software to be used in the project

#### Corresponds to DMSP Elements: “Related Tools, Software, and/or Code”

The DMSP Elements recommends providing *“an indication of whether specialized tools are needed to access or manipulate shared scientific data to support replication or reuse*, *and name(s) of the needed tool(s) and software”* [10]. This requirement accompanies and complements the requirements for sharing information about project data because knowledge of the tools and software used in the project supports reproducibility, which is an underlying motivation of the Final NIH Data Management and Sharing Policy. Reproducibility is “*the ability of a researcher to duplicate the results of a prior study using the same materials as were used by the original investigator*. *That is*, *a second researcher might use the same raw data to build the same analysis files and implement the same statistical analysis in an attempt to yield the same results”* [16]. Data is just one part of the original materials used in a study; the software and tools used to gather and manipulate the data are equally important. Data scientist and reproducibility expert Victoria Stodden emphasizes the importance of computational reproducibility or providing information about the code, scripts, hardware, software, and implementation details of a study in order to enable full reproducibility, allowing for the integral part that computers and software play in modern science [17].

In a compliant DMSP, describe the following:

- Devices that will be used to collect project data.
- Software or programming languages that will be used to work with the data (e.g., Python, STATA, R).
- Whether the tools and software are open source (free) or proprietary (must be purchased).
- If known, note how long the tools and software will be usable to access the data (e.g., until a software program’s end-of-life date).

### Rule 4: Use standard file types, identifiers, and descriptive elements

#### Corresponds to DMSP Elements: “Standards”

The third section of the DMSP Elements guidance asks the researcher to provide *“an indication of what standards will be applied to the scientific data and associated metadata (i*.*e*., *data formats*, *data dictionaries*, *data identifiers*, *definitions*, *unique identifiers*, *and other data documentation)*,*”* including explications of any common data standards used [10]. Multiple aspects of data can be described under the topic of standards, and many aspects of data mentioned under standards in the guidance are covered in earlier sections like “Data Types.”

The standards requirement of the NIH DMSP asks researchers to outline, to the extent possible prior to the start of the project, where standardization will be implemented that will ultimately make the data more accessible for future secondary uses. Firstly, describe whether standardized file types will be generated, such as open-source types (e.g., a CSV file used over a proprietary Excel spreadsheet). In addition, if data dictionaries will be employed to define variables, it would be appropriate to outline briefly the dictionaries’ standard format.

Employing unique identifiers is also recommended in the standards section. This refers to online persistent identifiers, or PIDs, which are long-lasting references to a digital resource [18]. These identifiers can be assigned to any person, organization, or concept, and their linkable nature is the foundational concept behind linked open data on the web. PIDs allow machines to identify and gather resources based on semantic concepts, just as human-readable metadata terms allow for human search and retrieval of resources.

A DOI or digital object identifier is perhaps the best known and most robust persistent identifier for digital outputs of any kind, including datasets. DOIs for scholarly articles, for example, help to track their impact online. A DOI is a special, long-lasting type of URL that is assigned to a digital resource by an identifier registry such as DataCite or CrossRef. This assignment generally happens automatically when a digital resource is deposited into an institutional or other type of digital repository. Compliance with this aspect of the DMSP requirements can be achieved by noting the intention to preserve data in a repository that assigns DOIs. Alternatively, other PIDs in wide use in biomedical research can also be utilized to identify and locate online datasets, these include NCBI accession numbers, PMCIDs for small datasets submitted as supplementary materials to articles in PubMed Central, and Ensemble or Genome identifiers.

If additional standardized documentation, such as controlled vocabularies, are planned to be applied to data from the beginning stages of the project, this can be noted briefly in the DMSP as well. Some standards may be in use as part of daily work, such as an ORCiD (an identification number serving to disambiguate researchers), and others might be encountered occasionally throughout the research process (such as the Medical Subject Headings or MeSH). Identifiers commonly used for outputs, people, and concepts in research workflows, which conveniently also incorporate PIDs, are shown in Table 1. Utilizing these types of identifiers when describing data for sharing helps to make data FAIR (findable, accessible, interoperable, and reusable). Data described and preserved according to the FAIR principles is maximized for interoperability and machine readability, which in the long term enables increased impact, discoverability, and computational access to data [19].

**Table 1:** Commonly used PIDs in research workflows.

**Outputs:** • Digital object identifier (DOI): permanent URL (uniform resource locator) or hyperlink that will always lead users to the resource, even if its home webpage is renamed or changes. • NCBI accession numbers: unique identifiers for sequence records [20].
**People:** • ORCID ID: Per their website, ORCID is “an international, interdisciplinary, open, nonproprietary, and not-for-profit organization created by the research community for the benefit of all stakeholders, including you and the organizations that support the research ecosystem” [21]. Individuals who sign up with ORCID receive an http URI with 16 digits that uniquely identifies them. Appending this URI, or PID, to research outputs will help with citation counts and other metrics.
**Concepts (examples):** • Medical Subject Headings (MeSH): subject terms with associated PIDs for biomedical concepts [22]. • UniProt names and taxonomy section: provides information about protein and gene names, as well as the organism that is the source of the protein sequence [23]. • Other unique persistent identifiers, such as those outlined in the PID Graph [24].

Keep in mind that full data description examples employing all the abovementioned standards do not need to be provided along with a compliant DMSP; however, if there is an intention to employ such standards, this may be stated and further plans to utilize such standards can be incorporated later into an implementation plan.

In addition, if no consensus standards exist that can be applied as metadata or descriptors for the project’s data, it is acceptable to note the lack of consensus standards in the DMSP.

### Rule 5: Understand the options for preserving data

#### Corresponds to DMSP Elements: “Data preservation, access, and associated timelines”: Point 1

The fourth section of the DMSP Elements guidance requires the researcher to list *“the name of the repository(ies) where scientific data and metadata arising from the project will be archived”* [10]. A repository, or digital online storage system for data, is an important choice, as repositories can differ drastically from each other, and in order to be compliant with the DMS Policy must meet certain requirements for the accessibility of the data.

The NIH provides an additional guidance document on *Selecting a Repository for Data Resulting from NIH-Supported Research* to make the process of choosing a repository simpler and more streamlined [25]. In a decision-tree-like manner, repository selection recommendations are presented as follows:

1. If the NIH and/or Institute, Center, Office (ICO) policy(ies), and Funding Opportunity Announcements (FOAs) require use of particular repositories as listed in their documentation, use the required repositories.
2. If there is no NIH ICO-determined repository, use an established repository that is appropriate for the project’s data type(s) and that is vetted within the respective research community
  - As a subset of the above, preference should be given to domain- and data type-specific repositories. The NIH lists such repository examples on its Open Domain-Specific Data Sharing Repositories webpage [26].
3. If there are no domain- or data type-specific repositories, the NIH recommends using generalist repositories [27], institutional repositories, or submitting small (less than 2 GB) datasets as supplementary materials along with a publication contribution to PubMed Central.

### Rule 6: Find a repository

#### Corresponds to DMSP Elements: “Data preservation, access, and associated timelines”: Point 1

If one has followed the repository selection narrowing process from Rule 5 and has determined that either an institutional or one of the many existing generalist repositories must be utilized for depositing data, it can still be challenging to know whether the repository is appropriate or whether it fulfills the NIH’s requirements for data sharing. The NIH’s *Selecting a Repository for Data Resulting from NIH-Supported Research* contains recommendations (“Desirable Characteristics for All Data Repositories”**)** on repository characteristics that can help with this decision [25]. Repositories meeting these criteria simultaneously meet the majority of criteria for making data FAIR as defined by the international GoFAIR, stakeholder-driven initiative [19].

The “Desirable Characteristics” should be reviewed carefully by the research team, especially members of the team with data management expertise, before a repository is listed in the DMSP. If a more appropriate repository is identified later based on the characteristics, an update to the DMSP is warranted. The “Desirable Characteristics” are detailed, but are summarized here in the following categories:

- **Metadata and PIDs:** A unique identifier such as a DOI is assigned to the data deposit by the repository; in addition, descriptive metadata fields in the repository enable FAIRness, utilize vetted schemas, and enable citation.
- **Easy access:** Free access for de-identified data records having no pre-existing restrictions; data reuse is enabled through clear licenses; the repository employs common, preferably nonproprietary formats. Guidance on how to use data is clear.
- **Long-term sustainability:** The repository has a long-term management plan and retention policy.
- **Curation/provenance:** Repository either provides or allows access to people providing curation or quality control assistance for the creation of data deposit records.
- **Security/integrity/confidentiality:** Repository’s levels of security match the sensitivity of the data. There is documentation noting security levels, confidentiality protections, and risk management protections.

The NIH’s *Selecting a Repository* guidance contains further requirements for repositories that store human data, even if it has been de-identified [25]. Review these more stringent requirements as well and make sure they are met, if needed, as part of the repository selection process.

The NIH has recognized the role that generalist repositories play in enabling data discovery and reuse [28] and has published a non-exhaustive list of generalist repositories to serve as a guide for repository identification [27]. Institutional repositories (including InvenioRDM and Dataverse) and generalist, publicly available repositories (e.g., Zenodo, Dryad, and others participating in the NIH Generalist Repository Ecosystem Initiative [29]) all serve as support for biomedical data reuse by enabling long-term, discoverable FAIR data deposits.

Institutional repositories and generalist, publicly available repositories serve many researchers’ data preservation needs, while simultaneously serving the needs of those seeking datasets for reuse within the prescribed limits and licenses. Institutional repositories are maintained by researchers’ institutions and are often maintained by the institution’s library. Such tools have buy-in from the host organization and generally have plans for long-term support. Institutional repositories also often have the added benefit of the availability of support staff within the institution to help with data ingestion and metadata creation.

As an addition to the NIH’s guidance, it is helpful to know of repositories that can support creation of a metadata-only record. This type of record does not require the deposit of a data file. Such records are key to sharing information about human subjects data, since these datasets can be difficult to de-identify and share through a repository. By creating a metadata-only record that *represents* datasets containing personal health information (PHI) or personally identifiable information (PII), the datasets become discoverable, notifying others to the existence of the data, access to which can be brokered via a “Contact the Researcher” feature in the repository or via email contact. Through this method of sharing, sensitive data are maintained by the original researcher and shared on a case-by-case basis after a Data Use Agreement is completed.

### Rule 7: Coordinate timelines for data sharing

#### Corresponds to DMSP Elements: “Data preservation, access, and associated timelines”: Point 3

Timeliness of data sharing is specifically addressed in the DMSP Elements guidance. While data sharing is recommended at the release time of an associated publication or at the end of the performance period, whichever comes first [10], there are often additional timelines to consider for data sharing based on additional parties’ interests in the data. Like funders, journals frequently have requirements to share portions or all of the data within specific time frames based on project completion or article publication date. Examples of requirements for data sharing by journals are seen in author guidelines by publishers Science and PLOS, both of which require sufficient data to allow replication of the experiment or analysis at the time of article publication [30,31]. Other timing factors include: institutional or award-based requirements for records retention or, the long-term preservation of data records of note [check institutional records management policies and the FOA’s retention requirements], patent-able aspects of the data and whether limitations are placed on data due to its support of novel inventions, and time required to adequately de-identify human subjects data. If plans for data preservation and archiving, cleaning for the purposes of sharing, and applicable patents are coordinated, data preservation person-hours can be maximized.

Such varying stakeholder timelines have significant effects on the total time frame for data availability. Different subsets of the data may need to be made available at different times; Gantt charts and other project management or scheduling tools can help to manage these timelines at the time of DMSP implementation. Making data available for its expected useful lifespan is another factor to plan for and outline in the DMSP [10]. At the time of submission of the DMSP, list the anticipated times for data sharing based on stakeholder requirements as far as they are known at the time. These timelines can be updated as part of regular updates to the DMSP throughout the project lifecycle. As a best practice, consider the retention guidelines of the federal government (generally 3 years after the completion of the grant/submission of the final financial report [32,33]), followed by institutional retention requirements, then those of the funder and potential publishers. Plan to preserve data for at least the length of the longest mandated retention period.

### Rule 8: Protect privacy and confidentiality prior to access

#### Corresponds to DMSP Elements: “Access, distribution, or reuse considerations”: Part 1

As an agency funding biomedical research studies, the NIH enumerates in their DMSP Elements guidance the various factors potentially limiting research data availability. While it does promote maximum appropriate sharing of data, it acknowledges that this must be done with strict attention to privacy, security, informed consent, and proprietary concerns [10]. Privacy and security concerns begin at the point of data collection.

The human subjects data and specimens collected through clinical research contain a wealth of identifiers, from personal information and vital statistics to tissue samples. In a new *DRAFT Supplemental Information to the NIH Policy for Data Management and Sharing*: *Protecting Privacy When Sharing Human Research Participant Data*, the NIH enumerates principles for protecting patient privacy as well as strategies for data de-identification [34]. Consulting the standards of the Common Rule [35] and the Health Insurance Portability and Accountability Act (HIPAA) Privacy Rule [36], is recommended, such as the Safe Harbor (removing all 18 identifier types) and Expert Determination (employing the assistance of a person with sufficient scientific and statistical knowledge to render the data unidentifiable) methods. Either method should be vetted by attempting to re-identify individuals using advanced computational methods. While the primary investigator is responsible for any data de-identification strategies, institutional data security experts should also be consulted to review the de-identification plan and final de-identified datasets before publicly sharing data.

Any de-identification strategy must also respect federal, tribal, state, and local laws and regulations for maintaining data derived from human subjects. A good place to start to explore state laws is the HealthIT.gov website, which contains listings of state consent and patient permission laws [37]. Likewise, a place to start for information on collaboration with tribal communities with respect to data access is the NIH’s DRAFT Supplemental Information to the NIH Policy for Data Management and Sharing: Responsible Management and Sharing of American Indian/Alaska Native Participant Data [38].

Informed consent of the participants in studies utilizing human subjects data is another factor with vital implications for data sharing. To help comply with increased funder calls for data sharing, many academic health centers’ institutional review boards (IRBs) are updating their requirements for informed consents to include sections on potential present and future sharing of the collected data. In addition, the NIH has recently published a resource on informed consent language to facilitate future data sharing [39]. It is good practice to outline in consent documents any immediate plans for sharing data related to funders and imminent publications, as well as any plans to deposit datasets to a repository for sharing with collaborators or future, unknown researchers. This disclosure provides the patient the ability to opt out of the study if they are not comfortable with this level of data sharing or with the idea that their data may be aggregated, pooled, or reused on new studies far into the future. The language used in the consent forms should outline clearly the exact levels of data sharing to which the patient agrees upon providing their consent. For instance, if such is the case, it should be clearly stated that patients will not be contacted or re-consented for future sharing or accessing of their data through repositories.

### Rule 9: Know the controls and approvals needed for data accessibility

#### Corresponds to DMSP Elements: “Access, distribution, or reuse considerations”: Part 2

Per the DMSP Elements guidance, the NIH requires descriptions of how access to the data might be controlled by or dependent on approvals [10]. Data accessed or licensed from other sources, as in the case of secondary and meta-analyses, should only be made available if the original source permits data sharing. Similarly, Data Use Agreements (DUAs), which govern the transfer of data between institutions, can have a wide-ranging effect on individual researchers’ data sharing capabilities. Specifically, DUAs must be in place per the HIPAA Privacy Rule before the collaborators can share limited datasets. Such limited datasets typically have some identifiers removed, but may contain indirect identifiers such as city or zip code, age in years, and dates such as visit dates, birth date, etc. [40].

For the participants in a DUA, the potential restrictions on data sharing in order to protect the privacy of study participants are clear. Data should not be further disclosed beyond the ways permitted by the agreement, and when disclosed via these ways, the data recipient must apply safeguards to prevent unauthorized usage and disclosures. If the DUA allows data sharing, it is key for all participants’ DMSPs to specify the permitted venues for sharing (e.g., institutional repositories), the data’s visibility level (will it be shared only with in-house researchers or with the general public?), and which additional de-identification measures will be taken to ensure that a subset of the limited dataset can be deposited or shared as a truly anonymized dataset.

Any additional legal, regulatory, or policy-based restrictions imposed on data must be taken into account before data are shared. Consider the variety of restrictions that may exist over the lifecycle of data ownership and processing. Secure the appropriate permissions and maintain them with the project’s documentation.

### Rule 10: Plan for and outline oversight of data management and sharing

#### Corresponds to DMSP Elements: “Oversight of data management and sharing”

The sixth recommended section in the NIH’s DMSP Elements guidance requires researchers to indicate how compliance with the plan will be monitored, with what frequency, and by whom [10]. Since science involves many people on a daily basis, including one or more principal investigators (PIs), co-investigators, post-docs, graduate assistants, and interns who collect data, biostatisticians who help to construct analysis plans and conduct analyses, data analysts who create complex database queries, and more, it may be challenging to imagine coordinated data management and compliance accomplished by only one or a few people.

While PIs are ultimately responsible for data management, PIs are increasingly leveraging the skills and expertise of specialized information professionals to fill a dedicated data manager role. The research team’s data manager is responsible for overseeing data as it moves from collection or querying to analysis, storage, and sharing, all while ensuring data integrity and protection of research subject privacy.

The Contributor Role Ontology describes a data manager as “a role that encompasses effective and efficient operation and usage of data, including, but not limited to management, handling, or manipulation” [41]. If the analysis of data management practices resulting from the exercise of writing a DMSP demonstrates that a data manager could be a useful addition to the project team, it may be possible to employ evidence amassed from creating the DMSP to make the case to institutions or funders for data management support. Data manager qualifications will vary based on discipline and the types of data requiring processing and management. Ideally, a project data manager will be responsible for introducing, enforcing, interpreting, and regularly overseeing compliance with an NIH data management plan.

A data manager can get assistance and a great return on investment (ROI) from the DMSP creation process by making DMSPs machine actionable. The DMPTool, a free online resource providing templates for data management plans for various United States-based research funders, allows quick creation of compliant DMSPs [42]. While constantly accessible at the DMPTool website, the DMSP will be more relevant and connected to your study if it is made interoperable through the incorporation of key web-based standards for DMSPs. The first is the Research Data Alliance’s Common Standard for Machine-actionable Data Management Plans, a metadata model outlining several key attributes of research datasets, such as their creators, funders, host, and security and privacy requirements [43]. When included in a DMSP, these attributes can be further enhanced for the web by applying PIDs to as many of them as possible, including identifiers for creators, host organizations, and even methods and materials (by using ORCiDs, Research Organization Registry PIDs [44], and the Resource Identification Portal [45], respectively). The NSF in a recent Dear Colleague letter encouraged such efforts to make DMSPs machine-actionable, noting that machine interpretation of a DMSP can be a time saver in preparing repositories to receive datasets for ingestion [46]. If your DMSP is deposited in the same repository where your protocol, data files, READMEs, and other relevant study documentation is stored, be it an institutional or generalist repository, you will then have created an interoperable collection of study documentation that allows for maximum accessibility and reproducibility of your research [47].

Lastly, but not least, the NIH has outlined clearly in their *Supplemental Information to the NIH Policy for Data Management and Sharing*: *Allowable Costs for Data Management and Sharing* guidance that the costs associated with data curation, preservation, and management are allowable costs and can be factored into the budget justification [48]. These costs can be put toward dedicated team-based data managers or toward partial FTE time from academic libraries or other institutional departments with data management expertise.

## Conclusion: Considering stakeholder perspectives

While DMSP work may seem daunting, from the viewpoint of the many stakeholders in the research process, it is clear that the benefits of implementing and following improved data management practices and actively sharing data are worth the additional effort. Good practices can impact and benefit all stakeholders, including the research team, their library and other institutional stakeholders, the publishers who can play a role in dissemination of the work, and funders who support research to better understand ROI and meaningful impact. Good DMSP practices can support better engagement with and accountability to the public who benefit from research.

### Funders and publishers

Though this article specifically addresses the data management and sharing requirements of funded projects of the National Institutes of Health, this agency is not alone among US Federal agencies in seeking to increase data management best practices and sharing among awardees. Funding organizations and agencies in the US have sharpened their focus on data sharing over the past 10 years in response to calls for greater availability of the products of funded research made by their governing bodies. A 2013 memorandum from the White House Office of Science and Technology Policy (OSTP) required federal agencies supporting research to provide plans for increased public access to research data [49]. As each agency has worked to complete their plans, they have released guidelines to funded researchers outlining responsibilities for research data preservation and sharing. The NIH has encouraged some form of data sharing for projects funded at over $500,000 since 2003 or nearly 20 years.

Publishers require various levels of data sharing in accompaniment to submitted articles, a trend that promises to be on the rise in the future. Science, Springer Nature, Wiley, and Sage all offer guidelines on their websites for data sharing for submitted articles, and Taylor & Francis, Springer Nature, and PLOS require data availability statements, which let readers know where the data accompanying articles can be found if it is not included as supplemental material to the article itself [50]. The reproducibility crisis identified in science in recent years has been an impetus for these requirements as noted in stories of retractions and the inability to access older research data files to corroborate studies as the media and computing environments on which they were made and stored become obsolete [6,51]. The increase in fully online and open access journals is also helping to bolster the popularity of shared or repository-deposited data, as such dissemination methods allow for a linked network of digital objects that reinforce and support each other while supporting maximum reproducibility.

### Researchers and their host institutions

The benefits to the host organizations of researchers that accrue through implementing best practices in research data management and sharing cannot be overestimated. Simultaneously, the benefits of data sharing to individual researchers are continually proven as publication tracking and research impact assessments become more widely available. Researchers such as Piwowar [52] and Colavizza [53] have pointed out a connection between an increase in citation rates and sharing of datasets related to published resources. Researchers’ host organizations also benefit from the accumulated increase in citation counts from all its researchers. Likewise, its reputation benefits as more of its studies are proven reproducible as a result of effective management of data and code. Fewer retractions of articles are necessary when data has been well managed, which further increases institutions’ worldwide standing.

Institutions’ awareness of the importance of effective management and use of research data is reflected in the growing number of informatics positions supporting health science research. Advances in technology brought about and fostered by informaticians can lead to more effective tools for researchers, with better interfaces, quality control, and organization of terminology. In addition, the scientific methods practiced by informaticians themselves often involve meta-analyses, which require cleaned and normalized data [54].

### Libraries

Libraries may not be the first stakeholders to come to mind for researchers looking to manage data, as they may think of librarians as those who help gather resources for reviews or for project start-ups, rather than those who provide resources throughout the life of a project or who help plan for data preservation. Yet increasingly, librarians are gaining and offering the skills and services to assist researchers throughout the lifespan of their project, from data management planning and implementation, to training research staff in varying levels of data cleaning and preparation, in addition to support for best practices for long-term preservation. As Garcia and colleagues point out, “most biologists receive little or no formal preparation for the increasingly computational aspects of their discipline. In consequence, informal training courses are often needed to plug the gaps” [55]. Librarians are a growing force of professionals currently providing those training courses. Check with your institution’s library to see if they offer courses in basic data management, file organization, data cleaning with popular platforms such as Excel or OpenRefine, depositing materials to institutional and other repositories, and a wealth of other data management topics. In addition to training events, many librarians can work with researchers to upload their materials to repositories, either through basic assistance or through a service-based, mediated deposit.

The NIH DMS Policy presents an excellent opportunity for greater understanding and coordination of resources, stakeholders, and support on campus to meet (and potentially even exceed) the requirements. Researchers’ institutional libraries often provide an excellent starting point for assistance with data management and sharing best practices and can provide referrals to other units for additional support on specific facets of the policy. Colleagues, program and human subjects protection officers, and data-savvy units or cores on campus may also have recommendations to support compliance and help build and support efforts toward standardized data management.

### The public

Data management plans describe the practices around the data generated from research and ultimately how those data are preserved and shared. While DMSPs strengthen researcher workflows and research reproducibility, they also play a role in accountability and engagement with taxpayers who fund that research. Each year, the NIH budget is supported by federal appropriations (topping 41.6 billion dollars in 2020 [56]), ultimately made possible by taxpayers. The NIH’s investment of these funds in biomedical research brings significant returns, including catalyzing improved human health, driving the economy, generating new knowledge, advancing technology, and empowering a skilled biomedical workforce [57]. Improved practices to support sharing also support greater access to research data by the public and help enhance scientific accountability to the public—a responsibility of all stakeholders, including researchers, funders, institutions and their library, and publishers. The DMSP can provide a powerful vehicle for public engagement and understanding.
